# Advancing Drug Innovation for Neglected Diseases—Criteria for Lead Progression

**DOI:** 10.1371/journal.pntd.0000440

**Published:** 2009-08-25

**Authors:** Solomon Nwaka, Bernadette Ramirez, Reto Brun, Louis Maes, Frank Douglas, Robert Ridley

**Affiliations:** 1 Special Programme for Research and Training in Tropical Diseases (TDR), World Health Organization, Geneva, Switzerland; 2 Swiss Tropical Institute, Basel, Switzerland; 3 University of Antwerp, Antwerp, Belgium; 4 Ewing Marion Kauffman Foundation, Kansas City, Missouri, United States of America; McGill University, Canada

## Abstract

The current drug R&D pipeline for most neglected diseases remains weak, and unlikely to support registration of novel drug classes that meet desired target product profiles in the short term. This calls for sustained investment as well as greater emphasis in the risky upstream drug discovery. Access to technologies, resources, and strong management as well as clear compound progression criteria are factors in the successful implementation of any collaborative drug discovery effort. We discuss how some of these factors have impacted drug discovery for tropical diseases within the past four decades, and highlight new opportunities and challenges through the virtual North–South drug discovery network as well as the rationale for greater participation of institutions in developing countries in product innovation. A set of criteria designed to facilitate compound progression from screening hits to drug candidate selection is presented to guide ongoing efforts.

## Introduction

The discovery of novel drug leads with the potential to become usable medicines is an important component of the drug innovation cycle, but remains a major obstacle in the development of new drugs for infectious tropical diseases [1]. Historically, this innovation cycle starts with basic research followed by the translation of this research into product leads, their further development, and associated regulation, commercialization, and ultimate health impact [1],[2]. The process is long (typically 10 to 15 years), expensive (costing between US$500 and US$800 million according to pharmaceutical industry estimates), and technically challenging [3]–[5]. It should be mentioned that the cost identified above includes cost of failed projects, and the actual figure for neglected diseases will probably be significantly less.

Ideally, the interfaces between the various processes are well resourced and managed to ensure continuous availability of health products. Unfortunately, product research and development (R&D) and access is skewed in favour of ailments that are commercially attractive, while those diseases that disproportionately affect poor populations in developing countries are neglected. Consequently, an estimated 1 billion people, one in six of the world's population, currently suffer from neglected tropical diseases, mostly in developing countries [6]. The afflicted populations are poor and commercial incentives to enable investment in the risky product R&D are weak [3],[7]. The few available drugs have problems related to their cost, safety, stability, and increasingly, the threat of resistance that may limit their utility.

Some of the neglected diseases include malaria, trypanosomiasis, leishmaniasis, schistosomiasis, filariasis, and onchocerciasis. Malaria is a major health problem with over 1 million deaths occurring mainly in children and pregnant women in sub-Saharan Africa [8],[9]. New and affordable antimalarial drugs that are effective against resistant parasites and can be safely administered to children and pregnant women are urgently needed [3],[10]. Even for malaria, which has probably had significant investment, the drugs expected from the existing R&D portfolio in the next few years are a combination of old drugs that are mostly reliant on artemisinins. Going by the industrial assessment of attrition, the number of projects in the pre-clinical phase of R&D is too small to guarantee success. Recent reports of artemisinin resistance in Asia [11] means that emphasis has to be placed on the discovery of novel entities to support the medium to long-term needs of malaria control as well as possible elimination and eradication. The ongoing discussion on malaria eradication requires major technical and financial investment today to support the discovery and development of the tools needed for eradication beyond the next 15 years.

Tuberculosis (TB) therapy is made complex by the emergence of drug-resistant strains, and the long courses of treatment [12]. The treatment of human African trypanosomiasis (HAT) depends on drugs that are very toxic and requires intravenous administration. New drugs that are effective for both the early- and late-stage disease and have a greater ease of administration are highly desirable [13]. Therapy for Chagas disease is limited to nifurtimox and benznidazole, for which toxicity is dose-limiting [14]. Miltefosine and paromomycin have recently been approved for visceral leishmaniasis, but drugs for cutaneous disease remain toxic, difficult to deliver, and of marginal efficacy [15]. Treatment of diseases caused by parasitic worms (schistosomiasis, lymphatic filariasis, onchocerciasis) is dependent on a few drugs, many of which are suboptimal [16],[17]. A macrofilaricidal (adult worm-killing) drug is desperately needed for onchocerciasis and lymphatic filariasis control, as the drugs now in use require treatment throughout the 10- year lifespan of adult worms [18]. Moreover, drug resistance is now widespread in worms that affect livestock, and a similar emergence of resistance in human populations would severely hamper control efforts [19],[20]. There are also efforts focused on developing drugs including better antibiotic treatment regimens that can kill the *Wolbachia* endosymbiont of filarial parasites as a means of inhibiting embryogenesis and killing the filarial worms.

The past 10 years have witnessed a new emphasis on the promotion of innovation and investment in R&D for some of these diseases, which are largely supported with new funding from governments as well as philanthropic agencies, notably the Bill & Melinda Gates Foundation and the Wellcome Trust [3], [21]–[25]. Despite these developments, gaps still exist in the product supply chain for nearly all diseases that disproportionately affect the developing world. Recent analysis undertaken by some agencies, including the World Health Organization (WHO) Special Programme for Tropical Disease Research (TDR), as well as the BIO Ventures for Global Health [26], have identified critical gaps in the R&D process for some of these diseases, covering the following areas: a) translation of basic research to product leads to feed the development pipeline, b) product development for the most neglected diseases including the helminth infections, and c) implementation research or research to inform access and changes in drug policy.

With regards to translational research for lead discovery and development, we previously described an innovative drug discovery platform for infectious diseases of poverty that involves coordinated networks and partnerships with industry and academia in both the developed and developing countries, and how the networks might be scaled up to achieve a robust pipeline of new products for the neglected diseases [1],[27].

In this paper, we discuss advances and gaps in drug discovery for tropical diseases in the past few decades as well as factors that have contributed to recent progress in drug discovery for these diseases. Specific examples and lessons are drawn from the coordinated drug discovery network for multiple diseases [1]. A set of criteria designed to facilitate compound progression from screening to hit-to-lead and drug candidate selection for these diseases is shared with the hope that it will be useful for the broader discovery community for neglected diseases.

## Evolution of Drug Discovery for Neglected Diseases

The impetus for the development of some of the current drugs against tropical diseases was largely motivated by the needs of colonialism during the early 20th century [28], by wars in disease-endemic areas, and by animal health needs [29],[30]. The launch of the TDR in 1976 coincided with the time when pharmaceutical companies began to withdraw from the discovery and development of drugs for tropical diseases. By the end of the 20th century this withdrawal was almost complete [31]. The lack of commercial incentives to support the increasing cost of R&D for drugs against tropical diseases, coupled with the increasing stringent regulatory requirements, are sometimes cited as reasons for this withdrawal. This development prompted TDR's involvement in product development right from its inception through collaboration with R&D-based pharmaceutical companies to ensure that candidate drugs already in development are not shelved. Some of the success stories through those collaborations are well documented, for example with Merck in early 1980s over ivermectin for onchocerciasis, and with Bayer in the late 1970s over the development of praziquantel for schistosomiasis [31],[32].

During the 1980s, it became clear that the prospects of new chemical entities entering the development pipeline for tropical diseases were bleak. The high attrition rate also meant that the few remaining drug discovery programmes that continued within the pharmaceutical industry had limited chance of becoming registered products. Thus, this led to an increased focus on testing compounds already in development in companies for other therapeutic areas for potential utility in tropical diseases. Funding support from TDR enabled compound screening against tropical disease pathogens to be performed in public institutions such as the London School of Hygiene and Tropical Medicine and the Swiss Tropical Institute, as well as at Janssen Pharmaceuticals Belgium in 1993 and later at Tibotec Belgium. This approach markedly reduced discovery costs given the free access to compounds (meant for other purposes) provided by industry. The success achieved between 1985 and 1995 through this approach included the investigation of a potential treatment for visceral leishmaniasis and African sleeping sickness that resulted in the registration of miltefosine through a collaboration with Zentaris, and eflornithine through a collaboration with Aventis for those diseases, respectively [29],[33]. Both drugs were originally developed as anti-cancer agents. This piggy-backing or therapeutic switching had the potential of delivering new drugs more quickly and at lower costs since much of the development work had already been done [34]. However, many believe that the full power of innovation cannot be realized for tropical diseases through this approach.

Between 1995 and 2004, TDR expanded its screening activities to introduce the concept of a screening network for various tropical diseases with the added benefit that experiences, reagents, and standard operating procedures (SOPs) are shared among the partners. The activity further evolved to include the testing of compounds sourced from academic laboratories and commercially purchased compounds. This approach resulted in the evaluation of peroxides from different laboratories, including the University of Mississippi, the Universite Laval Montreal, and the University of Nebraska. Although TDR did not have the medicinal chemistry and drug metabolism and pharmacokinetics (DMPK) resources at that time to take forward promising hits and leads, it should be noted that some of the identified hits or leads helped some of the public private partnerships (PPPs) that emerged in the late 1990s and early 2000s such as Medicines for Malaria Venture (MMV) and Drugs for Neglected Diseases Initiative (DNDi) to rapidly identify and initiate lead optimization [3],[35],[36]. One example of such a lead optimization program is the Ozonide project supported by MMV that entered clinical development for malaria [37]. Some of these PPPs have realized the need to invest in the more risky early lead identification and are now extending their operations into this field.

Within the same period, several academic and public centres of excellence have emerged to support drug discovery for neglected diseases. For example, the Wellcome Trust drug discovery unit at the University of Dundee focuses on early stage drug discovery for HAT; the Sandler Foundation drug discovery facility at the University of California in San Francisco focuses on early drug discovery for Chagas disease, schistosomiasis, and malaria; and the Harvard/Broad Institute focuses on malaria. Prior to the establishment of these centres, the Central Drug Research Institute in Lucknow, India, and the Walter Reed Army Research Institute were also engaged in R&D for tropical diseases. It should also be noted that several institutions from developing countries are now emerging as possible centres of excellence in specific aspects of the drug discovery process. Examples include the National Institute for Parasitic Diseases in Shanghai supporting screens for schistosomiasis, the BIOTEC Institute in Bangkok supporting TB and malaria screens and chemistry, the University of Cape Town for malaria, and the National Institute for Pharmaceutical Research and Development in Abuja, Nigeria, for TB and malaria.

**Figure 1:**
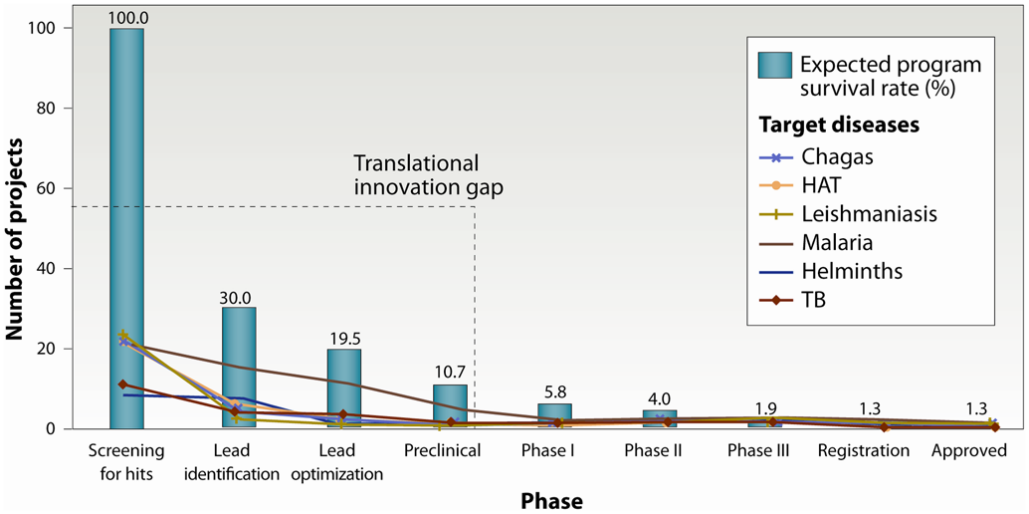
Attrition rates and current drug R&D pipeline for neglected diseases. The early-stage drug pipeline for neglected tropical diseases when compared with a typical industry-driven pipeline for diseases with commercially attractive indications illustrates a significant gap in the discovery and preclinical phases, referred to as “translational innovation gap”. Our current analysis is consistent with earlier reports [1],[25]. Assuming that the average industry attrition rates apply to projects in neglected diseases, the current screening, lead identification, and optimization programmes are significantly below what is required to yield a registered drug. This insufficiency leads to a “translational innovation gap” that needs to be urgently addressed to ensure the availability of new drugs for neglected diseases. (Sources: TDR, MMV, TB Alliance, and a number of academic institutions.)

During the past 4 years, the TDR drug discovery programme has evolved from simply performing compound screening to an integrated virtual drug discovery network that includes other parts of the discovery process, including target selection, medicinal chemistry, and DMPK activities, with the goal of identifying lead and drug candidates to sustain and feed the development pipelines. This network differs from other drug discovery initiatives by way of its broad disease scope, its distributed and virtual nature, its central coordination or management approach, the potential for spin-off of independent initiatives, and above all, the North–South and South–South capacity-building elements [1],[27]. These factors also illustrate the significant economies of scale achieved through the networks. The network activities have further evolved to include specific hit-to-lead as well as lead optimization projects driven by designated project teams within the larger network. Despite the progress being made by several agencies, including through PPPs as well as dedicated academic and industry activities, critical “translational innovation” gaps still remain (from screening for hits to lead identification and optimization) for all of these diseases (Figure 1). This analysis is based on the number of ongoing projects at each stage of the discovery and development process from the various PPPs involved in portfolio management and other organizations. Our data is consistent with an earlier analysis that led to the inclusion of lead discovery as a strategic area of work in the new TDR strategy (see [38]). The present study mimics the findings by the BIO Ventures for Global Health [26]; however, the disease scope in our study is broader and includes the activities of the North–South drug discovery network implemented by TDR.

The encouraging progress achieved through the innovative North–South drug discovery network has raised the interesting possibility for the emergence of independent initiatives from the network. Examples of such initiatives include the Helminth Drug Initiative and the African Network for Drugs and Diagnostics Innovation.

The Helminth Drug Initiative (HDI) was initiated in 2006 following recommendations from expert consultative meetings convened by TDR. An HDI Task Force has been established to support the implementation of agreed activities [39],[40]. The initial mission of HDI is to discover preclinical drug candidates for further development against schistosomiasis, onchocerciasis, and lymphatic filariasis. A major challenge in the search for new drugs targeting helminth diseases is the lack of robust *in vitro* biological test systems with a suitable sample throughput, i.e., >10,000 compounds per annum, as well as a lack of validated targets to support high-throughput screening (HTS) [39]. Unlike other neglected diseases such as malaria, and diseases caused by kinetoplastid parasites, there is no dedicated product development partnership focusing on helminth diseases.

A critical mass of competent investigators and R&D infrastructure exists in some developing countries, including countries in Africa. With additional new capacity being built by several agencies, more trained investigators will become available in the coming years [1],[41]. In the past 2 years, several postdoctoral fellows from developing countries have been trained in industry or academia located in both the North and South as part of the drug discovery network activities. Furthermore, ongoing collaborations with MerckSerono, Pfizer, and other network partners from the public sector are all contributing to this capacity building [1],[42]. The medicinal chemistry centre at the University of Cape Town, as well as the screening centres at the Theodor Bilharz Research Institute (TBRI) in Cairo and other institutions, have made remarkable progress in training African scientists as part of network activities. The University of Cape Town has progressed a hit-to-lead project into full lead optimization through the dedicated work of African scientists including *in vitro* absorption, distribution, metabolism, and excretion (ADME) support from the African Institute for Biomedical Science and Technology (AiBST) in Harare, Zimbabwe. The TBRI, the Kenya Medical Research Institute (KEMRI), and the National Institute for Pharmaceutical Research and Development (NIPRD) in Abuja, to mention a few, have screened thousands of small molecules and natural products against schistosomiasis, malaria, and tuberculosis with several hits identified. These successes have provided the impetus for the establishment of the African Network for Drugs and Diagnostics Innovation (ANDI) with the objective of promoting and sustaining an African-led R&D innovation platform by discovering, developing, and delivering new products for diseases that are predominant in Africa [43]. ANDI is also envisioned to support capacity building and research on traditional medicines through its activities. A strategic and business plan is now being developed as part of the mandate of the ANDI task force that was established following the inaugural meeting in Abuja in October 2008. The future plan is to extend similar innovation networks to Asia and South America. We believe that such regional networks could become self-sustaining in the medium to the long term and can contribute to socioeconomic development in the respective regions.

With the expansion and success of the TDR drug discovery network, several new challenges have become evident. In addition to managerial and financial challenges, compound progression is sometimes delayed due to the slow turnaround time of screening or DMPK data that are required to guide synthesis efforts and specific lead optimization activities. Mechanisms are continually being put in place within the network to overcome these challenges. These challenges and emerging opportunities within the respective networks are discussed in the following paragraphs.

### Drug Target Prioritization Network and Rational Drug Design

*De novo* discovery of new chemical entities, starting with target selection, validation, and HTS (real or virtual) against molecular targets in protein-based assays, has received a lot of attention in the past few decades, especially with its link to genomics [44]–[48]. The goal of such campaigns is to identify “hits” with defined modes of action for further assessment in whole parasite assays and *in vivo* disease models. Unfortunately, the results achieved to date through this approach in the area of antibacterials and antiparasitics have been minimal due to high attrition [1],[49],[50]. Hits emerging from recent target-based HTS campaigns at various public and private institutions (for example, a TDR-supported screen at the Walter and Eliza Hall Institute [WEHI] in Melbourne) have largely not shown good correlation between enzyme inhibition and whole cell activity [51]. The WEHI screen was performed with about 100,000 compounds against the following enzymes: *Trypanosoma cruzi* trypanothione reductase, *Trypanosoma brucei* farnesyl pyrophosphate synthase, *Plasmodium falciparum* pyrophosphokinase, and histone deacetylase [1],[36]. DNDi is taking some of the hits that emerged from the screens forward.

It must be emphasized that the validation status of most of the enzymes or proteins used for these screens is not clear. Understandably, the genetic validation tools available for some of the parasites is limited or even nonexistent, and chemical validation is yet to be implemented widely. It should be recognized that neglected diseases are only beginning to benefit from various HTS technologies implemented in the pharmaceutical industry for commercially attractive indications a few decades ago [27],[44],[46]. What is needed now is a collective effort to exploit the initial investment made by the international community in sequencing the genomes of the various parasites in the discovery of new treatments [52]–[57]. This has led to the establishment of the TDR Drug Target Prioritization Network, which has developed an open-source database of drug targets [58] covering multiple disease pathogens in support of target selection for rationale drug design [59]. This database could support the development of innovative *in silico* screening tools for infectious tropical diseases, including computational and structural approaches for the discovery of novel pharmacophores.

A recent drug discovery agreement between Novo Nordisk, the National Centre for Drug Screening (NCDS) in Shanghai, China, and TDR, whereby targets are selected through TDR partners and the TDR targets database for HTS campaigns, exemplifies the utility of the targets database [60]. This agreement also includes the training of African scientists at NCDS.

### Compound Screening Network

This network is composed of public institutions from both developed and developing countries identified through an open call and a competitive selection process, and funded by TDR [1],[36]. The network has promoted compound screening for neglected diseases since the early/mid 1990s by evaluating compounds from investigators around the world at no extra cost to the investigators. Until recently, however, most of the active compounds or hits identified were not followed up and in most cases only resulted in publications with little expansion of structure-activity relationships (SARs) [1]. The network now includes medicinal chemistry and DMPK networks (see below). The increasing number of compounds screened through the network coupled with the desire to improve the turnaround time for data necessitated the recent expansion of this network with new centres such as the University of Washington, Seattle, United States, for antiprotozoan screens and four other centres in developing countries, namely, the Central Drug Research Institute in Lucknow, India, for filarial screens; the University of Buea in Cameroon for onchocerciasis screens; the Kenya Medical Research Institute in Kenya for natural product–based antimalarial screens; and the University of São Paulo, Brazil, for screens against American trypanosomiasis. The SOPs used by the new centres have been reviewed and aligned with the broader network and new data is already emerging from these centres.

The screens implemented through the screening network range from *in vitro* whole parasite screens to *in vivo* animal testing against the various disease pathogens as well as cytotoxicity assays. In an effort to better understand and harmonize the different parasite strains used, TDR undertook an inventory of strains used within the network. It became obvious that same parasite strains are sometimes defined by different nomenclature in different laboratories and the phenotype of the strains are oftentimes not well defined. An inventory of some available parasite strains used by the network is presented in Table 1. This inventory may not be an exhaustive list of available parasite strains, but it provides clarification to the sometimes confusing nomenclature and drug sensitivity phenotypes for strains commonly used in drug discovery today. Hopefully this inventory will be useful for the broader neglected diseases drug discovery community.

**Table 1:** Some Parasite Strains Commonly Used for Compound Screening.

Target Pathogens	Parasite Strains	Drug Sensitivity Phenotype
**Chagas disease**		
*Trypanosoma cruzi*	Tulahuen LacZ, Clone C4^a^ (same as Tulahuen ß-gal; Tulahuen CL2; Tulahuen C4 LacZ; MHOM/CL/100/Tulahuen)	Sensitive to benznidazole, nifurtimox
**HAT**		
*Trypanosoma brucei brucei*	Squib427 ( = STIB795)^a^	Sensitive to suramin; reference drugs: melarsoprol, pentamidine
	STIB950^b^	Sensitive to melarsoprol, pentamidine and suramin; resistant to diminazene, isometamidium and quinapyramine
	GUTat3.1^b^	Sensitive to suramin; reference drugs: melarsoprol, pentamidine
	STIB345^a^	Sensitive to diminazene aceturate
*Trypanosoma brucei gambiense*	STIB754/130R^a^	Sensitive to melarsoprol, pentamidine and suramin
	STIB930^b^	Sensitive to melarsoprol, pentamidine and suramin
*Trypanosoma brucei rhodesiense*	STIB900^a^	Sensitive to melarsoprol, pentamidine and suramin
*Trypanosoma congolense*	STIB910 ( = STIB249)^b^	Sensitive to melarsoprol, pentamidine and suramin
**Leishmaniasis**		
*Leishmania donovani*	MHOM-ET-67/L82^a^ (same as MHOM/ET/67/HU3; LV9)	Sensitive to sodium stibogluconate and miltefosine
*Leishmania infantum*	MHOM/MA(BE)/67^a^	Sensitive to sodium stibogluconate and miltefosine
*Leishmania major*	MHOM/SA/85/JISH118^a^	Reference drug: sodium stibogluconate
	MHOM/SU/59/NEAL-P	Reference drug: sodium stibogluconate
*Leishmania mexicana*	MHOM/BZ/82/Bel21^c^	Reference drug: pentamidine
*Leishmania panamensis*	MHOM/PA/67/Boynton^c^	Reference drug: meglumine antimonate
**Malaria**		
*Plasmodium falciparum*	NF54^b^	Sensitive to all known antimalarials
	3D7 (derived from NF54)^b^	Sensitive to all known antimalarials
	K1^b^	Sensitive to mefloquine; resistant to chloroquine and pyrimethamine
	GHA^b^	Sensitive to chloroquine
	T23^b^	Resistant to chloroquine and pyrimethamine
	D6^b^	Sensitive to chloroquine, pyrimethamine, sulfadoxine and quinine; less sensitive to mefloquine
	W2^b^	Sensitive to mefloquine; less sensitive to chloroquine; resistant to quinine, pyrimethamine and sulphadoxine
	TM91C235^b^	Multidrug resistant
	RCS^b^	Multidrug resistant
	FCR3^b^	Sensitive to pyrimethamine; resistant to chloroquine and cycloguanil
	TM90C2b^b^	Less sensitive to mefloquine, resistant to chloroquine and atovaquone
	WR87 (wild type)^b^	Not known
	KN27^b^	Not known
*Plasmodium berghei*	ANKA^c^	Sensitive to chloroquine and artemisinin
	N^c^	Sensitive to chloroquine
*Plasmodium yoelii*	NS^c^	Resistant to chloroquine
*Plasmodium chabaudi*	AS^c^	Resistant to pyrimethamine
*Plasmodium vinckei*	Not known^c^	Sensitive to chloroquine
**Lymphatic filariasis**		
*Brugia malayi*	India^a^	Reference drug: diethylcarbamazine
*Brugia pahangi*	Not known	Reference drug: diethylcarbamazine
**Onchocerciasis**		
*Onchocerca gutturosa*	Ghana^a^	Reference drugs: amocarzine, ivermectin and melarsomine dihydrochloride
*Onchocerca lienalis*	UK^a^	Reference drugs: amocarzine, ivermectin and melarsomine dihydrochloride
*Onchocerca ochengi*	Cameroon^b^	Reference drugs: amocarzine, ivermectin and melarsomine dihydrochloride
**Schistosomiasis**		
*Schistosoma mansoni*	Puerto Rican^a^	Reference drug: praziquantel
	Egyptian Sambon^a^	Reference drug: praziquantel
*Schistosoma haematobium*	Egyptian^a^	Reference drug: praziquantel

a Used for both *vitro* screens and *vivo* rodent models.

b Used for *vitro* screens.

c Used for *vivo* rodent models.

The screening centres communicate with each other and share lessons, SOPs, and reagents as well as data. Depending on need, multiple centres within the network can be engaged to perform *in vitro* or *in vivo* screens for similar or different pathogens. This practice avoids over reliance on one centre and also gives the additional advantage of enabling cross validation of data within the compound screening network. A potential disadvantage is the possibility to create redundancy and undue competition within the network. However, these concerns have not in any way hindered progress within the network. A central facility for compound management and databases help to overcome some of these challenges including communications within the network.

Compounds are sourced by TDR and distributed to the respective centres from a central compound management facility. The decision as to which compounds are to be screened by any of the centres is made by TDR. This decision is made with due consideration to ongoing screens, available capacity at a particular centre, and the type of compounds available for distribution. For instance, compounds with some animal health rationale are initially dispatched for anthelminthic screens. Once the *in vitro* screens are completed, the next challenge becomes the analysis and prioritization of actives especially for a large compound collection. This time-consuming prioritization of hits is perhaps one of the current bottlenecks for screens for neglected diseases—it includes closer assessment of chemical structures and drug likeness of all the hits before reaching a decision on further evaluation. For the North–South network, this task is implemented with the help of experienced consultants with industry experience recruited by TDR.

An analysis of the compound screening throughput at the various centres shows that approximately 10,000 compounds were evaluated against whole parasites in 2004–2005 compared with over 30,000 compounds in 2006–2007. This significant increase in the number of compounds reflects the substantial compound availability through TDR and its partners, including Pfizer, Chemtura, MerckSerono, and others. This has also resulted in some screening centres reaching their maximum capacity for the past 2 years. In addition, several hundred of the *in vitro* hits resulting from the ongoing prioritization exercise have been evaluated in *in vivo* animal tests and potential leads are continually being evaluated and considered for further optimization. To be clear, the compound screening network is primarily involved in *in vitro* (low to medium throughput) cell-based screens as well as *in vivo* animal parasite screens, and not the high-throughput target-based screens discussed earlier. However, the network is continually seeking funding opportunities to improve the throughput of available assays for tropical diseases.

### Medicinal Chemistry Network

The medicinal chemistry network was established by TDR in 2005 with the objective to take forward the hits emerging from the screening activities through individual hit-to-lead and lead optimization projects. This network includes public institutes and pharmaceutical companies from developed and developing countries selected through a competitive call for applications. The present membership of this network includes the University of Cape Town, South Africa; University of Dundee, United Kingdom; University of Nebraska, US; Ohio State University, US; St. Jude Hospital, Memphis, US; Pfizer, MerckSerono, Chemtura, and Pharmacopeia. A recent addition to the network is the University of São Paulo, Brazil, which is now working on a hit-to-lead project for compounds that are active against *Trypanosoma cruzi*, the pathogen that causes Chagas disease. As part of the capacity-strengthening component, a local *in vitro/in vivo* screening centre has been identified to support this project and two local postdoctoral trainees have started working on the project.

A variety of screening hits from different sources—industry, academia, and commercial suppliers—can enter the hit-to-lead and lead optimization process through the medicinal chemistry network once appropriate contractual agreements (materials transfer or technical services agreements) are reached with the respective centres. Issues contained in the agreement range from ownership/intellectual property rights on compounds, analogues, and data generated for infectious tropical diseases to the need for publications. The initial phase of the agreement typically focuses on lead or drug candidate discovery, with the understanding that if the results of the work provide reasonable indications that one or more of the compounds may be useful in the treatment of any of the diseases of interest, then the parties will enter another agreement on the further development of the compound(s) and to make the product available to the public, especially the public sector of developing countries, under preferential pricing.

A challenge that is emerging with select public institutions within the medicinal chemistry network is the tendency for some of these centres to over-value their contributions even when they are aware that the compounds they are working on and the original ideas behind the project, including funding, are not entirely coming from their institutions. This stems from the desire by these partners to have full ownership of intellectual property and control all results without consideration for the contribution of colleagues from other parts of the network, but most importantly without due consideration to the need for future identification of downstream development partner(s) if the compound survives the harsh attrition in the discovery phase. Resolving this issue is not always simple but obviously requires a strong leadership to ensure that the participation of different parties in the network is primarily driven by public health outcomes.

A select example of data from commercially available compounds acquired by TDR that formed the bases for the establishment of the medicinal chemistry network is presented below.

## TDR 17516—Hit-to-Lead Project for Malaria

**Figure 2:**
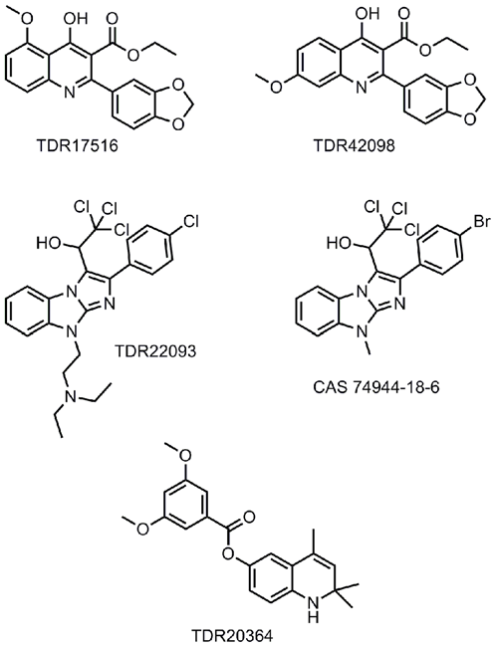
TDR17516 and analogue TDR42098; TDR 22093 and analogue CAS 74944-18-6; and TDR 20364.

A screening campaign commissioned by TDR at Tibotec using 17,472 non-proprietary compounds sourced from SPECS resulted in the identification and confirmation of the antimalarial activity of a compound subsequently coded TDR 17516 (Figure 2).

The resulting data include *in vitro* IC_50_s (ug/ml) against the following *P. falciparum* strains: K1 0.03 (compared with chloroquine 0.02) Selectivity Index (SI)>3000; NF54 0.0044 (compared with chloroquine 0.004); D6 0.0158 (compared with chloroquine 0.004); W2 0.058 (compared with chloroquine 0.084); TM91C235 0.049 (compared with chloroquine 0.046); and TM90C2B 0.082 (compared with chloroquine 0.08).

The *in vivo* data with mice infected with *P. berghei* include 97.7% inhibition of parasitemia at 4×100 mg/kg via the intraperitoneal (ip) route of administration; 91.3% inhibition at 3×50 mg/kg via ip route with 11 mean survival days (MSD) (≤7 MSD for control), and inactive at 1×30 mg/kg ip. Although further amounts of TDR17516 could not be sourced, it was possible to acquire 12 analogues to develop some SARs. Thus, moving the methoxy group from the 5 position (TDR 17516) to the 7 position (TDR 42098; Figure 2) allowed activity to be retained against *P. falcip*arum K1 (IC_50_ 0.03 ug/ml). However, moving it to 6 position (TDR 42099) results in a 10-fold loss of activity against the *P. falciparum* strain, whilst relocating it to the 8 position (TDR 42102) abolishes activity. Likewise, introducing alkyl substituents onto the N atom also resulted in loss of activity. Assessment of TDR 42098 in *P. berghei*–infected mice at 4×50 mg/kg ip showed no activity but no further material was available at the time. Additional analogues were needed to develop SARs further, plus more TDR17516 to complete assessment of efficacy in mice and to obtain a pharmacokinetic (PK) profile. A literature search failed to reveal published information on the lead (TDR17516), although the synthesis of numerous analogues have been reported but without claims of antimalarial activity. This series was assigned to the St. Jude Children's Hospital in Memphis for further chemistry in 2006 by TDR. In addition to the project idea and supporting data, TDR also provided funding support and consultant as well as ADME support through the Monash University in Australia and AiBST. It should be mentioned that the Swiss Tropical Institute generated additional biological data in support of the series transferred to St. Jude's.

## TDR 22093—Hit-to-Lead Project for Malaria

In an HTS campaign commissioned by TDR at Discovery Technologies Ltd., a non-proprietary compound library of 19,000 samples was screened against a plasmodial calcium-dependent protein kinase (Pf CDPK1). This resulted in a small number of hits (IC_50_ 1–10 uM) that were then tested against *P. falciparum*, culminating in the identification of one compound of interest, AE-848/08643022 (ex-SPECS), with moderate activity against the enzyme and whole parasite—approximately IC_50_ 2.88 uM. A search for related compounds was then made within the SPECS database and 38 analogues were subsequently sourced and tested against both enzyme and parasite. Although the enzyme activity was moderate with no compound having an IC_50_<10 uM, only one compound, AE-848/08581029, now called TDR 22093 (Figure 2), showed excellent activity against the *P. falciparum* K1 strain.

The *in vitro P. falciparum* IC_50_ (ug/ml) against K1 (Table 1) was 0.016 compared to chloroquine at IC_50_ 0.03 and SI∼400. The compound was also active against the NF54 strain.

Critical issues with the series included the need to synthesize further amounts of TDR 22093 to allow oral assessment against *P. berghei* and to determine ADME profile, and understand the SAR. In a search of the chemical literature, TDR 22093 was not found, although synthetic routes to close analogues such as CAS 74944-18-6 (Figure 2) were readily synthetically accessible (see structure above). This series was assigned to Pharmacopeia in the US for further medicinal chemistry and initial metabolism profiling in 2006 with initial funding from TDR. In 2007, MMV became a funding partner on the project, and this additional support helped to reach a “no-go” decision on this project within a year.

## TDR20364—Hit to Lead Project for Human African Trypanosomiasis

The same non proprietary compounds sourced from SPECS were tested against *Trypanosoma brucei* at Tibotec. From this initial assessment, TDR 15949 was identified with reasonable activity against *T. b. brucei* (IC_50_<0.5 uM). This compound contained the undesirable dinitrophenyl group, and subsequently a further 14 non-nitro analogues were sourced from SPECS and screened again. The data revealed only one compound, TDR 20364 (Figure 2), with significant activity in the Tibotec *T. b. brucei* assay. (Note: *T. b. brucei* is a cattle parasite used in the primary *in vitro* assay at Tibotec and in the primary *in vivo* screen at STI; in the latter it is coded as STIB 795 and is used because it is not refractory to treatment as STIB 900 is in mice.)

A re-test of the same compound at the Swiss Tropical Institute against *T. b. rhodesiense*gave even more encouraging activity with an IC_50_ 0.034 ug/ml. (Note: *T. b. rhodesiense* is a human parasite used in the primary *in vitro* screen at the STI and in the secondary *in vivo* assay in mice [strain coded STIB 900, cures are difficult to achieve].)

No further stocks of TDR 20364 were available for *in vivo* animal testing. However, a further 187 analogues were sourced from PrincetonBio of which 38 had IC_50_<0.05 ug/ml against *T. b. rhodesiense*. Many of these were simple ester variants of the lead compound. Two analogues, TDR 44218 (IC50 0.0075 ug/ml) and 44219 (0.038 ug/ml), were potent against the parasite and lacked the potential metabolic liability of an ester group. *In vivo* assessment in mice at 4×50 mg/kg ip against *T. brucei* STIB 795 showed an encouraging prolongation of life despite parasitaemia reduction being minimal with MSD>23.75 for TDR 44218 and 15.5 for TDR 44219, compared to 6.25 for control. Investigation of SARs showed that the majority of these compounds were close analogues of TDR 20364 with similar generic structure.

This series and analogues tested were assigned to Ohio State University for further chemistry in 2006. In addition to the project idea, TDR provided funding, medicinal chemistry consulting, and biology screening at the Swiss Tropical Institute and University of Antwerp, as well as ADME support through Monash University and AiBST. These network partners work collaboratively. A further SAR has been developed through this iterative medicinal chemistry work, and the project is now in lead optimization.

## Drug Metabolism and Pharmacokinetics Network

There is an increasing realization within the neglected diseases drug discovery community that early integration of DMPK in the drug discovery process is critical. Until recently, most drug discovery efforts for neglected diseases, especially those based in academic institutions, have focused on the synthesis of compounds with little guidance from parasite screening and DMPK data (*in vitro* and *in vivo*). This is understandable given that most academic laboratories have limited resources for carrying out advanced preclinical drug development.

Identification of quality leads requires extensive *in vitro* and a degree of *in vivo* ADME assessment aimed at guiding optimal chemical synthesis and SAR exploration (Box 2). The initial challenge in integrating DMPK into the drug discovery network was the identification of centres with the relevant capability. It was relatively easy to identify industrial partners already collaborating with TDR on compound supply and medicinal chemistry such as Pfizer, MerckSerono, Pharmacopeia, and Chemtura since a strong in-house capability for DMPK already exists within these companies. Interestingly, some of these companies have DMPK and toxicology data for some of the compounds supplied for screening. To be able to evaluate compounds from academic medicinal chemistry partners working on TDR compounds, the Monash University in Melbourne, Australia, and the AiBST, Zimbabwe, were identified. These two institutions have good track records and are already making excellent contributions to the activities of the entire network. However, with the increasing number of hit-to-lead projects within the medicinal chemistry network, it has become necessary to scale up the DMPK network with additional centres and resources to ensure the rapid turnaround of data to guide chemical synthesis.

A new development is that several external groups with hits or leads are now approaching TDR for DMPK support for their projects in a manner similar to support provided for compound screening over the years. Unfortunately, TDR has not been able to provide significant experimental DMPK support to projects that are external to the network due to limited resources. There is the possibility of using contract research organizations for early DMPK work, but issues like costs associated with repeated profiling of compounds, choice of initial assays, and continuity makes this option unattractive for some investigators.

### Network Coordination and Interface with Other Organizations

The success of any collaborative R&D effort largely depends on the management as well as the support available for the day-to-day implementation of the activities. The close interaction and interface between the individual networks is crucial in this regard. A regular joint meeting of the screening, medicinal chemistry, and DMPK networks has been implemented to help foster this close interaction, the sharing of data, and open discussion of issues relevant to all parties. In addition, individual hit-to-lead or lead optimization teams meet to discuss and address specific project needs within the network. An Expert Drug Discovery Advisory Committee (EDAC) also provides strategic review of the network projects annually. EDAC is composed of external experts from developing and developed countries with experience in the various areas of drug discovery, product development, public health, and parasitology. The EDAC review is based on a set of criteria for each of the networks. For example, the criteria for review of the screening centres include progress towards agreed milestones, the number of compounds screened and reproducibility of data, and the turnaround time of compound screens and data, as well as close interaction with chemistry and DMPK centres. The ideal turn around time is 4 weeks for *in vitro* screens and 2 months for *in vivo*. Similar criteria for medicinal chemistry, DMPK, and the targets network are also available. During the annual review, decisions are made about funding renewal for the individual projects; projects or centres that do not meet the expectation of the network are dropped. A recent review of the program by EDAC identified the need to strengthen the toxicological evaluation of promising compounds.

Due to the limited number of available experts in product R&D for neglected diseases, the network and indeed other R&D institutions are sometimes faced with the challenging task of identifying committee members. Committee members may sometimes have personal or institutional interests in projects or ideas being reviewed and evaluated. This potential conflict of interest is recognized and managed proactively. Participants at all meetings organized by WHO are asked to declare and sign a conflict of interest statement.

The North–South network model is not a panacea. A major part of the network coordination efforts is to ensure synergy and appropriate interface with other product discovery and development efforts for optimal impact. For example, the drug discovery centres at the University of California in San Francisco, the University of Dundee, the Swiss Tropical Institute, and the University of Antwerp, as well as institutions in the South such as University of Cape Town, the National Institute of Pharmaceutical Research in Abuja, Nigeria, and the Central Drug Research Institute in Lucknow, India, are all performing independent drug discovery research while also contributing to the drug discovery network activities. Some of the PPPs are also participating on specific hit-to-lead or lead optimization projects that have emerged from the network activities. For example, MMV is working with TDR on antimalarial lead discovery projects, while DNDi is working with the TDR network on some hit-to-lead projects for Chagas disease. Several biopharmaceutical, animal health, and specialty chemical companies, such as Pfizer, MerckSerono, Novo Nordisk, Bayer, Pharmacopeia, Scynexis, Chemtura, and Syngenta, are supporting the network in different capacities [1]. Some pharmaceutical companies have established dedicated drug discovery units for specific target diseases; for example, the GSK facility in Tres Cantos, Spain, focusing on malaria and TB, the Novartis Institute in Singapore focusing on TB, malaria, and dengue, and AstraZeneca in Bangalore and Eli Lilly in Seattle focusing on TB. These independent efforts are also encouraged to tap into parts of the network, for example, the TDR Target Database is an open-source database supporting global target selection which some of these initiatives are utilizing. Lessons learned through the screening network are broadly communicated for the benefit of other initiatives. Having said this, it should be emphasized that the pre-competitive but more open innovative nature of the North–South discovery network, as well as the disease scope, distinguishes this model from some of the other drug discovery initiatives. Although the present focus of this activity is lead and candidate discovery, it should be emphasized that strategies are being established to ensure that resulting drug candidates are promptly taken forward into development. One mechanism is to hand off leads to partners with the capacity for further optimization or development of such leads under appropriate contractual agreement, for example with PPPs or industry. Another mechanism supported by TDR that focuses on promoting innovation for product development in developing countries is now emerging as a viable option for hand off of leads or drug candidates to suitable centres or partnerships in developing countries. This latter approach is being strengthened through the establishment of regional networks exemplified by ANDI, as described earlier.

## Hit-to-Lead and Candidate Criteria

Current progress within the drug discovery network was, in part, made possible through the establishment of clear hit-to-lead and lead-to-drug candidate progression criteria covering the drug discovery process (Boxes 1 and 2). These progression criteria cover biological, physico-chemical, and pharmacokinetics, as well as early safety and toxicological components of drug discovery for various neglected diseases including malaria, African trypanosomiasis, Chagas diseases, leishmaniasis, lymphatic filariasis, onchocerciasis, and schistosomiasis [1],[3],[36],[46],[61]. The goal of presenting these criteria is to share our experience with various groups involved in drug discovery for neglected diseases with regards to the type of data required for compound progression and drug candidate selection. These criteria are not a substitute for specific target product profiles for each disease [1],[3], but rather it should complement the target product profiles as decisions are reached on compounds to progress into clinical development.

## Future Perspectives

Some may believe that promoting capacity building as an integral component of the product R&D process can distract from achieving the ultimate goal of discovering and developing drugs in a timely manner. We argue the contrary. Data now emerging from the North–South and South–South drug discovery networks show that human and institutional capacity can be built around drug discovery projects with clear product milestones and deliverables. Some of the medicinal chemistry, screening, and DMPK activities supported by young postdoctoral trainees in developed and developing countries have generated quality lead candidates. A recent example is the TDR 15087 hit-to-lead project for malaria at the University of Cape Town, which has progressed to lead optimization activities within 2 years. Although this project is supported by other TDR networks, most of the chemical synthesis has been performed by postdoctoral fellows from Africa, some of whom have already completed their training and have returned to their respective home institutions to start similar drug discovery projects. We believe that this trend will contribute to long term sustainability of access to essential medicines in disease-endemic countries.

The scalability of the network model can take the form of expansion of ongoing activities or spinning off parts of the activities depending on need or extending the model to other indications, including orphan diseases that occur in developed countries as well as the more commercially attractive indications. The more open innovation through the network does not threaten the creation or ownership of intellectual property [26], but rather may set the stage for an easy, cost-effective and more public health–centred approach for the discovery of novel medicines for various diseases, including antibacterials, antidiarrhoeals, and antiviral agents. The establishment of the African Network for Drugs and Diagnostics Innovation, whose objective is to promote and sustain African-led product R&D activities, is an example of a new initiative that can take on this challenge in Africa. We believe that stronger participation of the disease-endemic countries in the discovery, development, and delivery of the products they need will significantly contribute to ensuring long-term sustainability leading to the availability of health products for those countries. This emerging trend of promoting participation of developing countries in the innovation process needs broad support locally and internationally.

The innovation network activities described here are relevant to the WHO global strategy and plan of action on public health, innovation, and intellectual property [62]. This network covers parts of the eight elements of the global strategy and plan of action, which include: 1) prioritizing research and development needs, 2) promoting research and development, 3) building and improving innovative capacity, 4) transfer of technology, 5) management of intellectual property, 6) improving delivery and access, 7) ensuring sustainable financing mechanisms, and 8) establishing monitoring and reporting systems. Other existing mechanisms, such as PPPs exemplified by MMV, the Global Alliance for Vaccines and Immunisation, the International AIDS Vaccine Initiative, Foundation for Innovative New Diagnostics, the Global Alliance for TB Drug Development, and DNDi, should also be supported. Recent calls for applications announced by the Bill & Melinda Gates Grand Challenges [24], the Wellcome Trust [25], and some government agencies demonstrate the increasing appreciation that investment in R&D, capacity, and institutional development in disease-endemic countries will help to ensure sustainability in the longer term [1],[63]. We believe that equitable access and longer term availability of health products will be realized if we invest and promote R&D, and manufacturing within those countries.

**Box 1. Hit-to-Lead Identification Criteria for Protozoa and Helminth Diseases**

For clarity, a “hit” is a compound with selective *in vitro* activity (usually IC_50_<1 µM or expressed as ug/ml or appropriate unit) against whole parasite or enzyme or receptor, while a “lead” is a compound with basic drug characteristics conforming with the target product profile of a disease based on initial *in vitro* and animal data including efficacy, ADME, cytotoxicity, and chemical parameters. Progressing a hit to lead, and lead to drug candidate, requires a set of *in vitro* and *in vivo* efficacy, *in vitro* and *in vivo* ADME, cytotoxicity, and safety data, and physicochemical characterization. The acceptable baseline data required for lead and drug candidate declaration is summarized below and in Box 2.

**Hit-to-Lead Identification Criteria**

**Chemistry/Physicochemical Properties**

- Chemical structure confirmed and synthetic route established
- Good drug likeness index (e.g., ≤Lipinski Rule of Five violation for small molecules, no reactive entities in the structure)
- Compound could be reproducibly resynthesized to >90% purity
- Compound is chemically exploitable with regard to the potential for further SAR development and preferably novel
- IP situation clarified with no hindrance to exploitation for diseases of interest
- Predicted or measured physical chemical properties including aqueous solubility and permeability to demonstrate drug likeness for small molecules
- Indication of SAR pattern
- Natural product: structure of purified compound determined

**Biological and Initial Safety Data**

- *In vitro* activity confirmed against enzyme, protein, or whole cells
-
  - Antiprotozoan screens: IC50 and sensitivity index (SI) (ratio of L-6 IC50 and parasite IC50):
  -
    - *Plasmodium falciparum:* <0.2 µg/ml, SI>100
    - *Trypanosoma brucei rhodesiense:* <0.2 µg/ml, SI>100
    - *Trypanosoma cruzi:* <1.0 µg/ml, SI>50
    - *Leishmania donovani* or *L. Infantum:* amastigotes in macrophages 1–2 µg/ml, SI>20;
  - Anthelminthic screens:
  -
    - *Schistosoma mansoni:* 100% adult worm motility reduction, IC50<2 ug/mL
    - *Onchocerca lienalis* or *O. ochengi* or *O. volvulus:* 100% inhibition of microfilarial motility at 1.25×10−5 M or 10 ug/ml
    - *Brugia malayi:* 100% inhibition of microfilarial motility; 100% inhibition of adult worm motility and/or inhibition of MTT reduction at 10 ug/mL
- Determine selectivity over other related targets/parasites
- Demonstrate correlation between enzyme and parasite activity where the enzyme target of a compound is known
- Established selectivity for a molecular target or differential sensitivity between parasite and host enzymes should be >10-fold
- Active against resistant and sensitive strains (variation between strains is a warning sign)
- Acceptable pre-toxicity screening data in cellular screen and animals
-
  - Cytotoxicity (selectivity index)
  - Pre-toxicity screen in non infected mice using up to 100 mg/kg ip or po before *in vivo* efficacy studies
- *In vivo* activity usually in mouse or hamster models: significant reduction in parasitaemia and/or increase in life span, at 4×50 mg/kg either through the ip or po route with no overt sign of toxicity
-
  - *Plasmodium berghei* mouse model: >80% parasitaemia reduction & MSD of greater than that of untreated control (i.e., >7 days)
  - *T. b. rhodesiense* mouse model: 60 d aparasitaemia
  - *Trypanosoma cruzi* mouse model: MSD>30 days
  - *Leishmania infantum* hamster model or *L donovani* mouse model: *>*80% reduction in amastigote burden
  - *S. mansoni* mouse model: >75% reduction of adult worm load at 5×50 mg/kg
  - *Onchocerca lienalis/O. ochengi* mouse model: ≥75% reduction in microfilarial worm recovery at 5×50 mg/kg
  - *Brugia malayi* jird or Mastomys model: ≥80% reduction in microfilaria or adult worm recovery at 5×50 mg/kg
- Biological activity of single enantiomers determined if appropriate

**Metabolism**

- Metabolic stability determined in microsomes in at least two species including humans
- Preliminary exposure, ideally in the efficacy species, under conditions relevant to efficacy testing protocol

**Lead Selection Dossier**

- Preparation of a complete dossier containing an updated profile of compound and data accumulated so far
- Dossier reviewed and accepted by appropriate TDR consultants or committee

**Box 2. Lead Optimization and Candidate Selection Criteria for Protozoa and Helminth Diseases**

**Lead Optimization Criteria**

The goal is to develop further SARs around a lead to identify a short list of at least three candidates based on iterative medicinal chemistry to improve the intrinsic pharmacological properties of a lead, including activity in appropriate animal model, pharmacokinetics parameters, and exploratory toxicological assessments. The following criteria must be met to achieve this objective:

**Project Manager Assigned:**

- Work collaboratively to advance compound
- Identification of development partner and hand off approach clarified

**Chemistry/Physico-Chemical Properties**

- Criteria specific for oral (po) route of administration: aqueous solubility normally >1 mg/ml, logP<6
- Criteria specific to iv route of administration: aqueous solubility >1 mg/mL
- IP situation of compound clarified: require freedom to operate
- Preliminary assessment of chiral and diastereomeric purity if appropriate
- Chemistry amenable to synthetic analoging
- Process chemistry initiated for scale-up quantities (non GMP)
- Cost of Goods (COG) considerations highlighted

**Efficacy Data (*In Vitro* and *In Vivo* Activity)**

- IC_50_/Ki (dissociation constant for binding of inhibitor to an enzyme) against enzyme/receptor/target useful
- Whole organism activity IC_50_/IC_90_ versus a panel of laboratory strains—sensitive and resistant strains estimated
- Mode of action with regards to cidal or static useful to know
- Dose-related *in vivo* (rodent) activity<50 mg/kg established by proposed clinical route of administration (preferably oral) in comparison with appropriate reference compounds
- *In vivo* (rodent model) ED_50_/ED_90_ with acceptable pharmaceutical formulations in the predicted route for clinical trials
- Activity in a secondary assay if needed (e.g., central nervous system [CNS] mouse model like GVR35 strain for second stage HAT)
- Confirmed selectivity for the pharmacological target

**Metabolism**

- Metabolic stability in microsomes (intrinsic clearance in human and animal species)
- Relative ranking of potential to interact *in vitro* (as substrate or inhibitor) with human cytochrome P (CYP) 2D6, 3A4
- Acceptable CYP450 inhibition data

**Pharmacokinetics**

- po/iv pharmacokinetics in rodent species over therapeutic dose range may be useful
- Oral bioavailability targeted to a minimum of 20%
- Confirm *in vivo* systemic and/or tissue drug levels reach/exceed *in vitro* potency concentration
- Plasma half-life, Cmax, clearance, and volume of distribution in rodents

**Safety Pharmacology**

- hERG channel binding >10 µM (or dose escalation studies)
- Testing against a panel of G-protein coupled receptor (GPCR) and ion channel sites

**Decision:**

- Preparation of a complete dossier containing data accumulated so far
- Summary of all data in matrix with a comparator drug.

**Candidate Selection Criteria**

The goal is to perform additional studies to aid in the selection of a development candidate from about three or four top candidates for good clinical practice (GLP) pre-clinical development. Ideally, a candidate selection committee should be created to rank the candidates. In addition to data from lead optimization, the following criteria must be met to achieve this objective:

**Chemistry/Physico-Chemical Properties**

- Physical form of compound characterized log D, pKa, solubility, stability (tropical conditions). Salt form evaluated and most likely decided upon, and preclinical formulation(s) developed for use in PD/PK (pharmacodynamic/pharmacokinetic) determinations. COG estimate
- IP situation of compound: requires patentability
- Process chemistry initiated to scale-up to kg quantities (non-good manufacturing practice [GMP])
- Consideration for back-up candidates as appropriate

**Efficacy Data (*In Vitro* and *In Vivo* Activity)**

- Confirmed selectivity for the pharmacological target

**Metabolism**

- Major metabolites identified and characterized
- Enzyme induction potential; if necessary, test in human hepatocytes/suitable human cell line
- Evaluation of binding to major plasma proteins

**Pharmacokinetics**

- Full PK in at least three species including monitoring for major metabolites if possible, and allometric scaling for human dosing by the intended route of clinical administration

**Toxicokinetics**

- PK determined during the 5 days toxicity study (non GLP)
- Enzyme induction potential. If necessary test in human hepatocytes or suitable cell line.

**Toxicology and Safety Pharmacology**

- 5 day exploratory toxicology (2 doses) in rat and dog most typically (non GLP), including histopathology of major organs and evaluation of hemodynamic function and PK along the way
- Ames test with and without metabolic activation (non GLP)
- Micronucleus test in Chinese hamster ovary (CHO) cells
- Irwin test for CNS effects

**Decision:**

- Preparation of a complete dossier containing data accumulated so far
- Summary of all data in matrix with a comparator drug. The matrix must contain TPP for the disease and minimally acceptable drug criteria
- Dossier reviewed by TDR candidate selection committee
- Decision to pursue development by development partner
